# A retrospective cohort study on prevalence of postoperative complications in comminuted patellar fractures: comparisons among stabilized with Cannulated-Screw, Kirschner-Wire, or Ring-Pin Tension Bands

**DOI:** 10.1186/s12891-020-03936-5

**Published:** 2021-01-11

**Authors:** Xiao-zhong Zhu, Teng-li Huang, Hong-yi Zhu, Bing-bo Bao, Tao Gao, Xing-wei Li, Jun-qing Lin, Xian-You Zheng

**Affiliations:** Department of Orthopaedic Surgery, Shanghai Jiao Tong University affiliated Sixth People’s Hospital, 200233 Shanghai, China

**Keywords:** Patellar fractures, Kirschner‐wirecannulated‐screw, Ring‐pin, Tension band

## Abstract

**Background:**

Displaced patellar fractures are commonly treated with open reduction and fixation with several different types of tension-band (TB) constructs. The main objective of this study was to compare the prevalence of postoperative complications after surgical stabilization of comminuted patellar fractures with either a modified Kirschner-wire tension band (MKTB), a cannulated-screw tension band (CSTB), or a ring-pin tension band (RPTB).

**Methods:**

We conducted a retrospective and consecutive cohort study of comminuted patellar fractures (*n* = 334) stabilized using a TB construct. Postoperative premature loss of reduction, infection, and skin breakdown were compared according to the type of TB constructs received (MKTB, CSTB, or RPTB). The rate of implant removal due to symptomatic hardware was also evaluated.

**Results:**

Fixation failure rate was significantly different among the groups (*P* = 0.013), with failure rates of 4.7% observed in the MKTB group,14.5% in the CSTB group, and 4.9% in the RPTB group. Skin breakdown and infection were not significantly different among the groups (*Ps* > 0.05). Due to symptomatic hardware, 40.5% of the patients in the MKTB group, 22.9% in the CSTB group, and 24.3% in the RPTB group underwent implant removal (*P* = 0.004). After adjusting for age, gender, comorbidities, number of supplementary screws/K-wires, and use of cerclage cables, multivariate regression analysis revealed that CSTB contributed to a 2.08-times greater risk of fixation failure compared to RPTB, while MKTB and RPTB were similar in risk of failure. In addition, it was found that patients who underwent MKTB fixation were more than twice as likely to undergo implant removal for symptomatic hardware compared with RPTB (odds ratio = 2.11, 95% CI = 1.20 to 3.72; *P* = 0.010).

**Conclusions:**

RPTB have advantage over MKTB and CSTB fixation in terms of symptomatic hardware and premature failure, respectively.

**Level of evidence:**

Therapeutic Level III

## Background

Patellar fractures account for approximately 1% of all fractures in adults [1, 2]. Displaced patellar fractures are commonly treated with open reduction and internal fixation because of the important role the patella plays in knee function [3, 4]. The most widely adopted method of fixation is to use a tension-band (TB) construct, which converts the anterior tension force to a compressive force on the posterior surface [5, 6]. Different types of TBCs exist, such as modified Kirschner-wire tension band (MKTB), cannulated-screw tension band (CSTB), and ring-pin tension band (RPTB) [7–9].

Tension-band techniques provide good fracture healing results and functional recovery, but symptomatic hardware often leads to additional surgery to remove the implant [10, 11]. For transverse (C1) and stellate (C2) fractures, previous studies have concluded that CSTB is superior to MKTB, mainly because of fewer occurrences of symptomatic hardware for CSTB fixation, despite comparable failure rates [12, 13]. Likewise, in comparison of MKTB and RPTB fixation, one study showed that hardware-associated complications occurred at a decreased rate with RPTB fixation [14].

The surgical treatment of comminuted (C3) fractures is occasionally challenging, and fixation is more inclined to fail. A single TB construct is usually inadequate for comminuted fractures and therefore requires supplementary fixation. This includes stabilization with interfragmentary screws, Kirschner wires (K-wires) and cerclage wire/cables, which may enhance stability of the TB fixation [15].

The literature has very few studies reporting direct comparisons of different tension-band techniques in the treatment of comminuted patellar C3 fractures. One study, with approximately 50% of all included cases classified as C3 fractures, suggested there was a trend towards significantly more fixation failures with CSTB fixations compared to MKTB [16]. On the other hand, they also reported a decreased prevalence of symptomatic hardware with CSTB. In general, the evidence for superiority of one kind of tension-band fixation over another is equivocal for C3 patellar fractures, highlighting the need to clearly identify which technique has the lowest risk of both fixation failure, and symptomatic hardware. Thus, the main objective of the present study was to compare the prevalence of postoperative complications after surgical stabilization of comminuted patellar fractures with MKTB, CSTB, or RPTB.

## Methods

This retrospective study was approved by the Ethics Committee of Shanghai Jiaotong University Affiliated Sixth People’s Hospital. Informed written consent was obtained from all patients in accordance with the Declaration of Helsinki.

Patients treated at our hospital from March 1, 2016, to March 1, 2017, for C3 patellar fractures and who underwent surgical stabilization with a TB construct were identified as candidate participants (*n* = 504). C3 patellar fractures were classified according to the Orthopaedic Trauma Association/AO (OTA/AO) Classification [17]. Exclusion criteria were the presence of open fractures (*n* = 33), the presence of concomitant fractures (*n* = 45), loss of follow-up within one year after surgery (*n* = 37) and declining to participate (*n* = 55). All data for the 334 included patients were extracted from medical records.

Patient-specific factors have a direct effect on the prevalence of complications of surgically treated patellar fractures. For example, a history of cerebrovascular accident (CVA) and type 2 diabetes mellitus (T2DM) contributes to an increased risk of infection and symptomatic hardware [18]. Therefore, patient age, gender, smoking status, history of CVA, and T2DM were recorded and used as analysis variables.

**Fig. 1:**
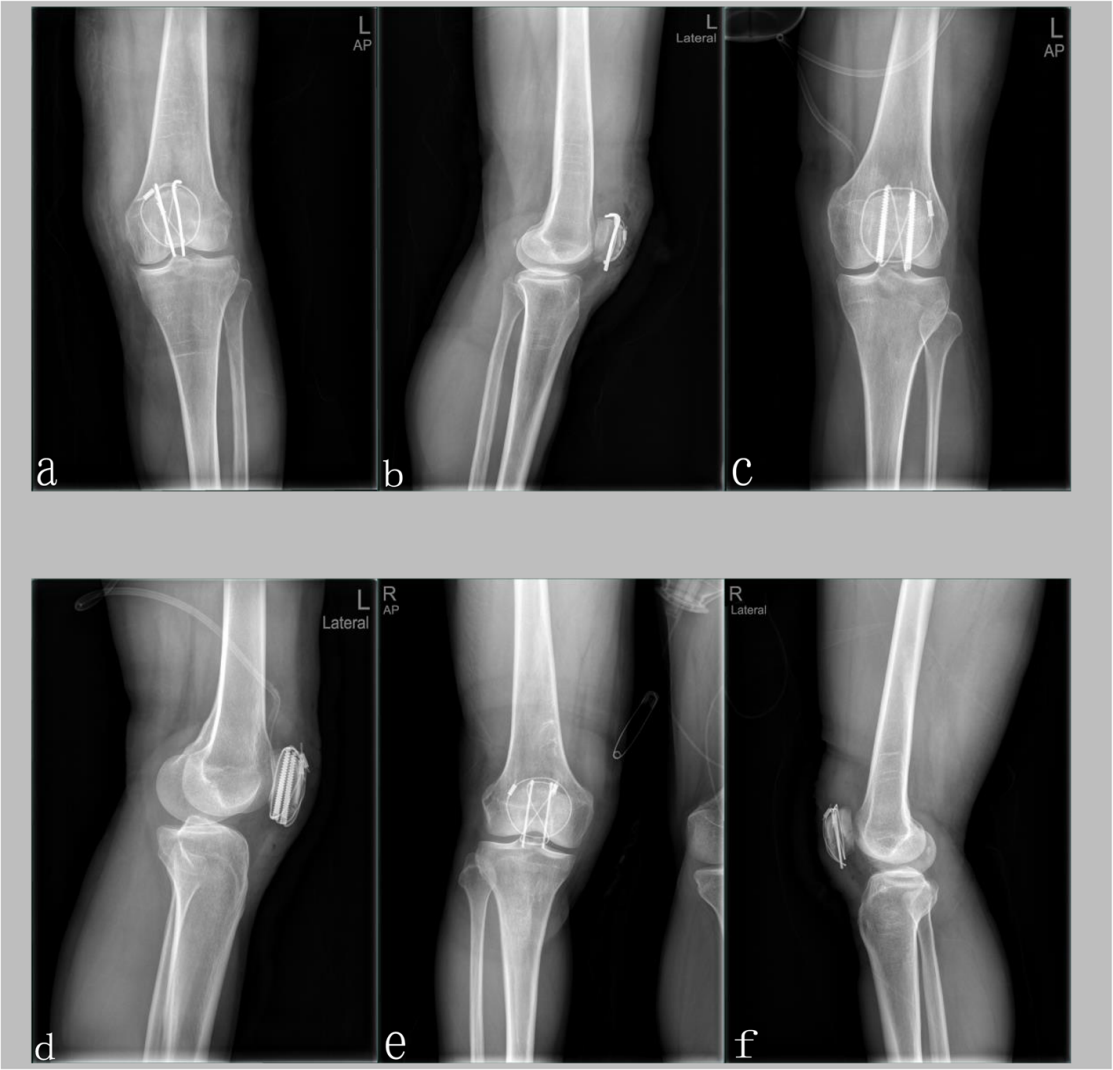
**a** AP radiograph of the knee, showing a comminuted patellar fracture fixed by MKTB. **b** On lateral radiograph, the articular step should be less than 1 mm. **c** AP radiograph of a case one day after undergoing fixation of CSTB. **d** The Lateral radiograph of the former case. **e** RPTB was used in this case. The tension bands were constructed in a figure-eight pattern. **f** The interfragmentary gap must be less than 2 mm

All surgical procedures were performed at a single hospital by 23 specialists in orthopedic trauma surgery, who followed the same treatment philosophy (achieving anatomic reduction of osteochondral fragments leading to stable osteosynthesis). Because there is no consensus on and reliable evidence for the optimal treatment of comminuted patellar fractures, the selection of MKTB (Fig. 1a and b), CSTB (Fig. 1c and d), or RPTB (Fig. 1e and f) was based on the treating surgeon’s preference. All tension bands were constructed in a figure-eight pattern with the use of titanium cables. Supplementary fixation, including interfragmentary screws or K-wires and cerclage titanium cables, was used if deemed necessary. None of the cases had an articular step greater than 1 mm or an interfragmentary gap greater than 2 mm on postoperative radiography. All patients received a single dose of a first-generation cephalosporin antibiotic for prophylaxis unless they were allergic. For patients allergic to cephalosporin, the type of antibiotic used depended on the surgeon’s preference.

Postoperative rehabilitation was similar for all participants but not entirely identical because of the complexity and variability of C3 patellar fractures. All patients performed quadriceps femoris contraction exercises immediately after the surgery. Active and passive knee-motion exercises were allowed and encouraged within one week after surgery. One month after surgery, patients were permitted to perform partial weight-bearing walking. Braces were not used in this study.

Premature fixation failure was defined as loss of reduction before the union of the fracture, thus requiring revision surgery. Indication for revision surgery was an articular step of greater than 2 mm, as determined by radiography. Implant breakage or loosening without secondary displacement of the fracture was not considered to be a failure. Postoperative infection was defined as an infection that occurred before wound healing. Skin breakdown was defined as a secondary skin lesion after the initial wound healing.

Continuous variables were presented as means ± standard deviation; categorical data were presented as numbers and/or percentages. When yielded P value was less than 0.2 in univariable analysis, the variable was further included in logistic regression for multivariable analysis. All statistical analyses were conducted using SPSS 22.0 (IBM Corp. Released 2013. IBM SPSS Statistics for Windows, Version 22.0. Armonk, NY: IBM Corp.). All statistical assessments were two-sided and were considered significant at p < 0.05. Differences among groups were assessed using Pearson’s Chi-squared test or one-way ANOVA, unless indicated otherwise.

## Results

A total of 334 patients with comminuted patellar fractures (OTA/AO C3) were included. MKTB(148 out of 334, 44.3%), CSTB(83 out of 334, 24.9%), or RPTB(13 out of 334, 30.8%) fixations were used for stabilization. Demographic and clinical characteristics are summarized in Table 1. The three groups were statistically indistinguishable on all these variables. None of the demographic variables were associated with fixation failure, implant removal, infection or skin breakdown (i.e., fixation failure, postoperative infection, skin breakdown, or implant removal) (all *P* > 0.05).

**Table 1:** Demographic and Clinical Characteristics of 334 Study Participants, Stratified by Fixation Type^a^

Characteristic	MKTB (*n* = 148)	CSTB (*n* = 83)	RPTB (*n* = 103)	*P* value†
Mean Age (*yr*)	53.7 ± 13.3‡	51.9 ± 13.9	52.5 ± 13.6	0.602
Gender				
Male	63 (42.6)	32 (38.6)	41 (39.8)	0.816
Female	85 (57.4)	51 (61.4)	62 (60.2)	
Comorbidities				
T2DM	33 (22.3)	18 (21.7)	30 (29.1)	0.380
History of CVA	5 (3.4)	3 (3.6)	5 (4.9)	0.828
Smoking	30 (20.3)	17 (20.5)	25 (24.3)	0.722

^a^Data are presented as the number of patients, with the percentage in parentheses, unless otherwise noted. †One-way ANOVA. ‡Standard deviation. *MKTB* modified Kirschner wire tension band; *CSTB* cannulated screw tension band; *RPTB* ring pin tension band. *T2DM* type 2 diabetes mellitus; *CVA* cerebrovascular accident

The mean fracture healing time was 2.21 months, which was not significantly different among type of fixation method (*P* > 0.05, assessed by one-way ANOVA). Overall, fixation failure occurred in 24 patients (7.2%) after the initial fixation (Table 2). Fixation failure varied significantly across fixation type: 7 patients (4.7%) of the MKTB group, 12 patients (14.5%) of the CSTB group, and 5 patients (4.9%) of the RPTB group experienced failures (Table 2, *P* = 0.013). Multivariate regression analysis, adjusting for age, gender, comorbidities, number of supplementary screws/K-wires, and cerclage cables (Table 3), revealed that CSTB contributed to a 3.08-times risk of fixation failure compared with RPTB (odds ratio = 3.08, 95% confidence interval [CI] = 1.01 to 9.43; *P* = 0.049). By contrast, the failure rates of MKTB and RPTB were similar (odds ratio = 0.94, 95% CI = 0.29 to 3.06; *P* = 0.913).

**Table 2:** Clinical Outcomes, Stratified by Fixation Type*

Characteristic	MKTB (*n* = 148)	CSTB (*n* = 83)	RPTB (*n* = 103)	*P* value†
Mean healing time(*mo*)	2.20 ± 0.31	2.22 ± 0.32	2.21 ± 0.33	0.902
Mean follow-up (*mo*)	18.4 ± 4.7‡	19.0 ± 4.6	19.2 ± 4.7	0.371
Number of supplementary screws/K-wires				
0	99 (66.9)	45 (54.2)	73 (70.9)	**0.006**
1–2	46 (31.1)	28 (33.7)	28 (27.2)	
3 or more	3 (2.0)	10 (12.1)	2 (1.9)	
Cerclage cable	136 (91.9)	78 (94.0)	95 (92.2)	0.839
Post-Operative Complications				
Fixation failure	7 (4.7)	12 (14.5)	5 (4.9)	**0.013**
Infection	14 (9.5)	5 (6.0)	7 (6.8)	0.584
Skin breakdown	4 (2.7)	2 (2.4)	3 (2.9)	0.978
Implant removal	60 (40.5)	19 (22.9)	25 (24.3)	**0.004**

*Data are presented as the number of patients, with the percentage in parentheses, unless otherwise noted. †One-way ANOVA. ‡ Standard deviation

**Table 3:** **Multivariate Regression Analysis of Fixation Method Failures, Controlling for Confounding Variables**^a^

	Odds ratio	95% CI	*P* value
Fixation failure			
MKTB (reference: RPTB)	0.94	0.29–3.06	0.913
CSTB (reference: RPTB)	3.08	1.01–9.43	**0.049**
Implant removal			
MKTB (reference: RPTB)	2.11	1.20–3.72	**0.010**
CSTB (reference: RPTB)	0.82	0.40–1.68	0.589

^a^Controlling for patient age, gender, comorbidities, number of supplementary screws/K-wires used, and use of cerclage cable

Skin breakdown and infection were not significantly different among the groups (*Ps* > 0.05, Table 2). Due to symptomatic hardware, 40.5% of the patients in the MKTB group, 22.9% in the CSTB group, and 24.3% in the RPTB group underwent implant removal (*P* = 0.004, Table 2). After multivariate regression, it was found that patients who underwent MKTB fixation were more than twice as likely to undergo implant removal for symptomatic hardware compared with RPTB (odds ratio = 2.11, 95% CI = 1.20 to 3.72; *P* = 0.010). The difference in prevalence of implant removal between CSTB and RPTB groups was insignificant (Table 3).

This study has two major end points. RPTB and MKTB have advantage in terms of premature failure over CSTB fixation while RPTB and CSTB had less rate of symptomatic hardware compared with MKTB. Thus, we could show the advantages of RPTB for the both end points at the same time by choosing it as reference.

## Discussion

For comminuted patella fractures fixation ,RPTB fixation is superior to CSTB fixation and MKTB in lowering the failure rate and it hardware removal respectively.

Complex three-dimensional forces, including bending, tensile, and compressive forces, are exerted on the patella as the fracture heals. This set of forces can lead to implant failure or loosening with continued patella-femoral motion [19]. As a result, significant rates of fixation failures in the face of early rehabilitation protocols are widely observed [16, 20–22]. For transverse and stellate fractures, previous studies demonstrated that fracture fixations using CSTB or MKTB have comparable failure rates [12, 13]. Laboratory biomechanical studies even show that the CSTB method has advantages over the MKTB method in fixation of transverse patellar fractures [5, 23, 24]. Notably, in clinical studies, comminuted fractures are more frequently encountered than transverse and stellate fractures. AO Type 34-3C was the most common patella fracture type,representing 2% of all fractures [25]. Most biomechanical studies have used the transverse osteotomy model instead of models that more realistically mimic comminuted fractures, limiting their clinical relevance. In our hospital, the selection of MKTB, CSTB, or RPTB fixation method is based on the treating surgeon’s preference because there is no consensus on and reliable evidence for the optimal treatment of C3 patellar fractures. To the best of our knowledge, our results reported here represent the first evidence from a large clinical study for the superiority of the RPTB over CSTB and MKTB method for fixation of comminuted patellar fractures.

Hoshino et al. observed a trend toward fewer fixation failures with the use of MKTB compared to CSTB for nearly half of the fractures classified as C3 fractures [16]. They concluded that the trend toward more fixation failures in the CSTB group might have met statistical significance if more patients were available for study. In the present study, we focused on evaluating the three different tension-band techniques in treatment of comminuted patellar fractures. Our results partially confirm the trend reported by Hoshino et al. [16]. In the present study, failure rate significantly increased with CSTB fixation of comminuted fractures compared with use of MKTB and RPTB methods.

With CSTB fixation, primary pressure is achieved by the two cannulated lag screws, [26] which are supplemented with a titanium cable; this bolsters the fixation through the cannulated screws. Unlike cannulated screws, ring pin and K-wires do not exert compression forces on the fragments directly. Instead, the compression effects on the patella are mainly generated by tightening the wires or cables [27]. In addition, the flexibility of K-wires and ring pins is another important difference compared with cannulated screws. Again, further biomechanical studies on comminuted fracture models are urgently needed in order to clearly elucidate the mechanism underlying our observations. Finally, some authors have found that the appropriate insertion of cannulated screws is technically difficult, because screws that are too short provide inadequate fixation, while screws that are too long may abrade the cable, causing to its to break prematurely [28]. Regardless of the mechanism, however, our results suggest that orthopedic surgeons should choose RPTB fixation over CSTB fixation for comminuted patellar fractures, since we observed that CSTB fixation contributed to a 2.08-times greater risk of fixation failure compared with RPTB.

Symptomatic implant irritation is the most commonly observed complication following fixation of a patellar fracture. Although symptomatic implant irritation is not a serious complication, it does require a second surgery, which increases the overall cost and brings additional burden to the healthcare system and patient. Most inserted K-wires used in TBCs tend to protrude and loosen, especially after migration. This leads to more soft-tissue irritation than that caused by cannulated screws and ring pins. Ring pins are designed to prevent postoperative migration by locking the pin with a cable through the ring. In the present study, symptomatic implants in RPTB fixations occurred at a similar rate postoperatively as that in CSTB fixations (using low-profile cannulated screws). Given that postoperative complications, like skin breakdown and infections, were indistinguishable among the groups, taken together, we conclude that RPTB fixation is superior to CSTB fixation in lowering the failure rate and it also has advantages over MKTB in hardware removal.

We recognize several strengths and limitations of our study. It’s a large cohort and which involves more than 300 samples, while limitations still exist. First, a number of different orthopedic surgeons treated the patients in the study, probably differing some in skill level. Patients also underwent slightly different rehabilitation programs as a result of different treating physicians. This could have affected study outcomes, particularly fixation failure rate. Second, the allocation of patients to MKTB, CSTB, or RPTB fixation groups was not random, given the observational nature of this study. Different surgeons had different preferences for the three techniques. Although we adjusted each outcome for possible confounding variables through use of multivariate regression, there may still have been other related factors we did not consider. Third, because it is a retrospective study, the lack of clinical information such as ROM(Range of motion)and extensor function is a limitation. Finally, there’s no generally accepted definition of symptomatic hardware. The implant removal could be suggested when the patient feel comfortable and is unable to bear it. But the final decision may vary slightly according to the endurance of different patients. Taken together, the finding of the advantages of RPTB fixation should be further confirmed in randomized trials.

## Conclusions

RPTB have advantage over MKTB and CSTB fixation in terms of symptomatic hardware and premature failure, respectively.

## Data Availability

The data are available from the corresponding author upon reasonable request.

## Abbreviations

TB: tension-band
MKTB: Modified Kirschner-wire tension band
CSTB: Cannulated-screw tension band
RPTB: Ring-pin tension band
OTA/AO: Orthopaedic Trauma Association/AO
CVA: Cerebrovascular accident
T2DM: Type 2 diabetes mellitus
ROM: Range of motion

## Trial registration

Researchregistry5379. Registered 23 February 2020 - Retrospectively registered, https://www.researchregistry.com/browse-the-registry#home/.
